# Estrogen receptor α polymorphism is associated with dementia in a Brazilian cohort

**DOI:** 10.18632/oncotarget.27744

**Published:** 2020-12-15

**Authors:** Rafaella Papalino Lopes Magnago, Valerio Garrone Barauna, Bruna Ferro Brun, Ana Cristina Lo Prete, Aline Morgan Alvarenga, Luciene C. Gastalho Campos, Neuza Felix Gomes Rochette, Letícia Batista Azevedo Rangel, Rodrigo Alves Faria, Paulo Caleb Junior Lima Santos, Ian Victor Silva

**Affiliations:** ^1^Universidade Federal do Espirito Santo - UFES, Vitória, ES, Brazil; ^2^Universidade Federal de São Paulo, Escola Paulista de Medicina - EPM/Unifesp, São Paulo, SP, Brazil; ^3^Universidade Estadual de Santa Cruz – UESC, Ilhéus, BA, Brazil; ^4^Universidade São Judas - USJ, São Paulo, SP, Brazil

**Keywords:** dementia, elderly, estrogen receptor α, polymorphism

## Abstract

The growth of the elderly population is a worldwide phenomenon and it is associated with chronic diseases, including dementia. In this scenario, the present study aimed to evaluate a possible association of estrogen receptor α polymorphisms with dementia in a Brazilian cohort. The subject sample was divided into two groups, control (*n* = 105) and case (*n* = 73), according to analysis of two predictive dementia tests (MMSE and CDR). The genotyping for the *ERα* PvuII (c.454-397T>C, rs2234693) and XbaI (c.454-351A>G, rs9340799) polymorphisms were performed by polymerase chain reaction-restriction fragment length polymorphism. The *ERα* PvuII pp genotype was associated with higher odds ratio for dementia (OR = 3.42, 95% CI = 1.33–8.77, *p* = 0.01, in a model including covariates. A linear regression model identified significant associations of the *ERα* PvuII genotypes (independent variable) with CDR scale (dependent variable), β = 0.26 and *p* = 0.001. In conclusion, estrogen receptor α PvuII polymorphism is associated with dementia in a Brazilian cohort. This finding may be useful for the identification of a possible set of significant genetic and clinical biomarkers for better understanding pathophysiology, early diagnosis and management of dementia.

## INTRODUCTION

One of the most striking features of the current world´s demographic dynamics is the process of population aging, that is, the increase in the absolute number and percentage of elderly people in the population as a whole, which has occurred since 1950, but mainly during the 21st century. In an updated UN projection in 2019, the number of people aged 65 and over was 129 million in 1950, rising to 422 million by 2020 and expected to reach 2.5 billion by 2100. This represents a 19.1-fold absolute growth.

In relative terms, the elderly population (aged 65 and over) accounted for 5.1% of the total 1950 population, rose to 6.5% by 2020 and is expected to reach 22.6% by 2100 (a 4.5-fold increase in percentage from 1950 to 2100). According to the UN, in 2015 the world population of people over 60 years old reached more than 12.3% [1].

The Brazilian reality is not very different from the global trend, but the process of population aging in Brazil is even faster. According to a UN 2019 survey, the number of elderly Brazilians (aged 60 and over) was 2.6 million in 1950, rising to 29.9 million by 2020 and expected to reach 72.4 million by 2100. This represents a 27.6-fold absolute growth. However, due to the socioeconomic conditions of the country, this process is accompanied by an epidemiological transition with a higher prevalence of chronic degenerative diseases [1–3]. Among the chronic-degenerative conditions that afflict this “new” population, hypertension, diabetes mellitus, osteoporosis, atherosclerosis and dementia, in particular Alzheimer’s disease, stand out [4]. The diagnosis of Alzheimer’s disease and other types of dementia, which occur with severe cognitive loss, is based on the observation of clinical data. More robust and technological means of diagnostic assistance, already used in other countries, are more difficult and economically inaccessible. In clinical practice, many neurocognitive disorders have similar or overlapping symptoms, making differential clinical diagnosis difficult [5].

Biochemical and genetic studies are important for identifying markers to make diagnosis more assertive, for predicting risk of disease, and/or differential diagnosis between dementias. The presence of biomarkers, based on genetic alterations, is one of the most promising fields for the development of early diagnosis of dementia. Studies have identified many genetic variances and single-nucleotide polymorphisms (SNPs) that are associated with AD risk. Estrogen-receptor gene polymorphisms are possible candidates for AD susceptibility [6, 7].

In this scenario, the present study aimed to evaluate a possible association of estrogen receptor α polymorphisms with dementia in a Brazilian cohort.

## RESULTS

### General, clinical and genetic characteristics of participants

One hundred and seventy-eight (*n* = 178, mean age of 76 ± 8) elderly agreed to participate in the study. Table 1 shows general and clinical data of the control (*n* = 105) and case (*n* = 73) groups. We observed differences for the variables of age, education, dyslipidemia and workout among groups. The distribution of values for the CDR scale was 55 participants (30.9%) for 0; 50 participants (28.1%) for 0.5; 47 participants (26.4%) for 1; 17 participants (9.6%) for 2; and 9 participants (5.0%) for 3.

**Table 1:** General and clinical characteristics of the studied sample

Variable	Control group (*n* = 105) *n*/%	Case group (*n* = 73) *n*/%	*p* value
**Gender**, female	90 (85.7)	62 (84.9)	0.88
**Age**	73 ± 7	78 ± 8	**0.01**
**Body mass index**, (Kg/m^2^)	27.7 ± 4.5	26.4 ± 4.8	0.07
**Self-reported race/color**, white	52 (49.5)	33 (45.2)	0.85
**Education**, higher education	19 (18.1)	10 (13.7)	0.43
**Smoking**, yes	35 (33.0)	23 (31.5)	0.80
**Alcohol consumption**, yes	30 (28.6)	30 (41.1)	0.08
**Hypertension**	72 (68.6)	57 (78.1)	0.16
**Dyslipidemia**	50 (47.6)	51 (69.9)	**0.003**
**Workout**, yes	103 (98.1)	63 (86.3)	**0.004**

The frequencies of *ERα* rs2234693 C (correspondent to the p allele) and *ERα* rs9340799 G (correspondent to the x) alleles were 55% and 63%, respectively. The genotypic distributions for the rs2234693 and rs9340799 polymorphisms were not consistent with Hardy-Weinberg equilibrium (X^2^ = 15.6, *p* < 0.01 and X^2^ = 4.6, *p* = 0.03, respectively).

### Association of ERα polymorphisms with dementia and CDR

Tables 2 and 3 shows Odds Ratio (OR) values for dementia using different models, including *ERα* genotypes: without additional variables, with age and gender, and including APOE, education and dyslipidemia. The *ERα* PvuII pp genotype was associated with higher OR for dementia (OR = 2.55, 95% CI = 1.15–5.66, *p* = 0.02, in a model without additional variables; and OR = 3.60, 95% CI = 1.36–9.52, *p* = 0.009, in a model including covariates).

**Table 2:** Comparison of ERα genotype frequencies between control and case groups

	Control group (*n* = 101)	Case group (*n* = 47)	*p* value	OR (95% CI)
***ERα* PvuII (rs2234693) **, %				
PP+Pp	83.2	66.0	**0.02**	**2.55 (1.15–5.66)**
pp	16.8	34.0		
***ERα*** **XbaI (rs9340799)**, %				
XX+Xx	60.1	72.3	0.16	0.58 (0.28–1.24)
xx	39.9	27.7		

**Table 3:** Analysis of multiple logistic regression for dementia using different models incluing ERα genotypes

Model 1: genetic variant, gender and age.	*p* value	OR (95% CI)
***ERα*** **PvuII (rs2234693)**, pp genotype	**0.006**	**3.52 (1.44–8.57)**
Gender, female	0.06	2.77 (0.95–8.04)
Age, years	**< 0.001**	**1.11 (1.06–1.17)**
***ERα*** **XbaI (rs9340799)**, xx genotype	0.32	0.66 (0.29–1.49)
Gender, female	0.29	1.75 (0.63–4.84)
Age, years	**< 0.001**	**1.10 (1.05–1.17)**
**Model 2: genetic variant, gender, age, education and dyslipidemia.**	***p* value **	**OR (95% CI)**
***ERα*** **PvuII (rs2234693)**, pp genotype	**0.010**	**3.42 (1.33–8.77)**
Gender, female	0.18	2.12 (0.71–6.33)
Age, years	**< 0.001**	**1.12 (1.06–1.18)**
Education	0.28	0.51 (0.15–1.71)
Dyslipidemia	0.19	0.59 (0.26–1.31)
***ERα*** **XbaI (rs9340799)**, xx genotype	0.60	0.80 (0.34–1.87)
Gender, female	0.55	1.38 (0.49–3.88)
Age, years	**< 0.001**	**1.11 (1.06–1.18)**
Education	0.18	0.44 (0.13–1.47)
Dyslipidemia	0.14	0.55 (0.25–1.22)

Age was a variable with significant OR in all models (OR = 1.12, 95% CI = 1.06–1.18, *p* < 0.001). However, *ERα* XbaI polymorphism did not show significant associations with dementia in the studied groups. In a stratified analysis for gender, the pp genotype for the *ER*α PvuII variant remained significant for females (OR = 3.73, 95% CI = 1.39–10.06). For males, the sample size was too small for analysis.

Linear regression models identified significant associations of the *ERα* PvuII genotypes (independent variable) with CDR scale (dependent variable), in overall participants: β = 0.23 and *p* = 0.005 for a simple model and β = 0.26 and *p* = 0.001 for a model, including age and gender as covariates.

## DISCUSSION

Dementia is a polygenic multifactorial disorder determined by genetic, metabolic and environmental interactions. Within dementias, AD is the most common case, accounting for about 60% to 80% of cases [8]. Menopausal and postmenopausal hormone disorders have been associated with cognitive and affective disorders. Estrogen protects against oxidative stress, facilitates synaptogenesis and regulates neurotransmission in brain systems associated with cognition [9]. It has been described that the regional distribution of estrogen receptors (ER) within the brain is surprisingly similar to the geography of brain pathology in Alzheimer’s disease [10]. The effect of Estrogen receptor alpha (ERα) polymorphisms on cognitive functions occurs due to the interaction of the concentration of estradiol with the receptor [11, 12]. Thus, the estrogen/ER complex becomes an interesting candidate to discover the genetic background of dementia. The two most studied polymorphisms in the ERα gene are PvuII (rs223493) and Xbal (rs9340799).

In the present study, the ERα PvuII pp genotype was associated with higher OR for dementia. These findings corroborate other studies that found that polymorphism in ER alfa is associated with an increased risk of AD in Caucasian [13], Chinese [14], Japanese [15], UK [16] and European [17] populations.

However, ERα XbaI polymorphism did not show significant associations with dementia in the studied groups. Other studies did not find significant association between the ESR1 XbaI polymorphism and AD risk for any model in Caucasian, Asian [13] or European population [17].

Regarding incidence according to gender, a larger proportion of the female population was obtained in both groups. Studies performed in Latin America showed slightly higher rates for female participants in all age groups [18]. Similar rates have been reported in studies conducted in Europe [19], Los Angeles, India and China [20]. In the present study, the pp genotype for the ERα PvuII variant remained significant for female, as observed by Porrello et al. in an Italian female Alzheimer population [21]. These data are contrary to those found by [15], where the association was observed only in males [15]. However, the results for cognitive functions depends on which Xba1 polymorphism the woman carries [12].

Regarding the level of schooling of the individuals, the population with incomplete fundamental or fundamental education presented greater cognitive deficit. An inverse relationship exists between educational level and dementia [22]. The prevalence of dementia in Lima was 3.7% of individuals with more than 8 years of schooling and 15.2% of those who were illiterate [3]. By a univariate analysis, the study conducted in Catanduva/Brazil also revealed a higher incidence of dementia among illiterate people [18]. In Chile, two studies reported a higher prevalence of cognitive impairment and dementia in rural settings and in people with low educational levels: cognitive impairment was 5.6 times higher among adults with low educational levels (17.2%) compared to those with high educational levels [23, 24].

Dyslipidemia may be the result of genetic and dietary factors, and several lipid subgroups, including fatty acids (saturated or unsaturated), triglycerides, cholesterol and phospholipids, are altered in dementia patients [25]. Hypertriglyceridemia is the main dyslipidemia linked to metabolic syndrome, a syndrome that also includes obesity and insulin resistance. In fact, studies investigating the role of lipids in the brain identified abnormal lipid metabolism as an important pathophysiological process in the development of dementias [26, 27]. Increased lipid levels may affect important cellular functions involved in dementia, including cell membrane flexibility, reduction-oxidation potential, and Aβ aggregation [28]. Lipid metabolism is closely associated with APP processing, which results in increased production of Aβ [29]. This suggests that dyslipidemia may be a risk factor of extreme connection with symptoms of mental illness, explaining its diagnosis. The *APOE* gene is the main genetic risk factor for Alzheimer’s disease/demetia and, in addition, associated with dyslipidemias [30]. The *APOE* E4 allele is strongly associated with cited phenotypes. Thus, in the present study we did not focus on this genetic biomarker [31, 32].

This study has some limitations. For a better statement about this polymorphism, a larger and more heterogeneous cohort would be needed. In addition, coexisting multiple factors are important in pathogenesis changes. The diagnostic research relationships for potential risk factors for dementia versus current clinical application should take into account both genetic and longevity biomarkers with patients. Diagnostic and prognostic research can no longer be exclusively based on currently used clinical tests, but rather new diagnostic protocols that involve the relevant factors.

## MATERIALS AND METHODS

### Participants and study design

The Laboratory of Cellular Biology of Aging (LBCE), located at the Health Sciences Center of the Federal University of Espírito Santo (UFES), obtained approval from the ethics committee on human research (number 1,307,524). It is an exploratory, quantitative, descriptive study of a comparative nature, carried out in long-term partner institutions and the Third Age Cohabitation Center with a sample of 178 elderly people located in the greater Vitória/Espírito Santo region, Brazil.

Participants were screened for an anamnesis and two already validated predictive dementia tests (Mini Mental State Examination - MMSE and the Clinical Dementia Rating Scale -CDR) were applied. The participation of a caregiver accompanying the elderly was necessary. Through the data obtained in the screening, individuals were divided into 2 groups: control and case. The control group consisted of all the participants who did not present clinical predictive signs of dementia, while the case group had a clinical predictive sign of dementia as well as presence of positive CDR and MMSE tests.

### Genotyping

DNA was extracted from leokocytes of peripheral blood collected from each individual [33] The genotyping for the two polymorphisms in the *ERα* gene [PvuII (c.454-397T>C, rs2234693) and XbaI (c.454-351A>G, rs9340799)] was performed by PCR-RFLP (polymerase chain reaction - restriction fragment length polymorphism). The primers used in intron 1 region of the *ERα* were F 5′CTGTGTTGTCCATCAGTTCATC3′/R 5′CCATTAGAGACCAATGCTCATC3′ and had a fragment of 119 bp. For each PCR reaction 1× buffer, there were 3 mM MgCl 2, 0.4 mM of each dNTP, 0.4 mM of each primer, 2.5 units of Taq DNA polymerase, and about 50 ng DNA. For the RFLP, PvuII and XbaI were used according to manufacturer’s specifications and the restriction fragments were applied on 12% polyacrylamide gel. After fixation, the fragments were 78 and 41 bp for the PvuII and 88 and 31 bp for the XbaI. From 178 participants, we genotyped 148 samples.

### Statistical analysis

Continuous variables were presented as mean and standard deviation and categorical variables as frequencies. Chi-square and Fisher’s exact tests were used for comparing general, clinical and genetic characteristics among control or case groups, and Hardy-Weinberg equilibrium analysis. Student´s *t*-test was used to compare continuous variables according with groups. Multiple logistic regression models were used to assess the odds ratio (OR) for dementia according to *ERα* genotypes, in a recessive model. The covariates used in the models were: gender (female), age (years), education and dyslipidemia. The recessive model was chosen based on previous studies: *ERα* PvuII PP+Pp *vs* pp genotypes (c.454-397T>C, rs2234693) and *ERα* XbaI XX+Xx *vs* xx genotypes (c.454-351A>G, rs9340799) [13, 17, 34]. Linear regression models were performed for analyzing possible associations of the *ERα* genotypes as independent variables with CDR as a dependent variable. Statistical analysis was performed on IBM SPSS Statistics 20 software, with significance level of *p* ≤ 0.05.

## CONCLUSIONS

Estrogen E receptor α PvuII polymorphism is associated with dementia in a Brazilian cohort. This finding may be useful for identifying a possible set of significant genetic and clinical biomarkers for a better understanding of pathophysiology, for diagnostic accuracy, differential diagnosis, early diagnosis, and treatment of dementias.
